# Case Report: Coexistence of Rosai-Dorfman disease and B-cell acute lymphoblastic leukemia in an adolescent

**DOI:** 10.3389/fped.2025.1529833

**Published:** 2025-02-26

**Authors:** Alireza Jenabzadeh, Fariba Binesh, Amir Pasha Amel Shahbaz, Samin Alavi

**Affiliations:** ^1^ Hematology and Oncology Research Center, Shahid Sadoughi Hospital, School of Medicine, Shahid Sadoughi University of Medical Sciences, Yazd, Iran; ^2^ Department of Pathology, Shahid Sadoughi University of Medical Sciences, Yazd, Iran; ^3^ Department of Radiology, School of Medicine, Shahid Sadoughi University of Medical Sciences, Yazd, Iran; ^4^ Pediatric Congenital Hematologic Disorders Research Center, Research Institute for Children’s Health, Shahid Beheshti University of Medical Sciences, Tehran, Iran

**Keywords:** acute lymphoblastic leukemia, Rosai–Dorfman disease, concurrent, bone involvement, liver involvement, dural involvement, children

## Abstract

Rosai–Dorfman disease (RDD) is an unusual, non-malignant proliferative disorder involving non-Langerhans cell histiocytes, characterized by a wide range of clinical presentations and distinctive atypical morphological patterns. The concurrent manifestation of acute lymphoblastic leukemia (ALL) alongside RDD is exceptionally rare. Here, we present the case of a 14-year-old male patient diagnosed with ALL who, during the consolidation phase of chemotherapy, developed multifocal bone, dural, and liver lesions, as confirmed through CT and MRI imaging. Histopathological evaluations of the bone and liver lesions identified features consistent with Rosai–Dorfman disease. To the best of our knowledge, this case represents the first reported instance of RDD co-occurring with high-risk pre-B-cell ALL in an adolescent undergoing chemotherapy. Unfortunately, the patient experienced a relapse of ALL and died due to a fungal infection. In this report, we analyze the distinct clinical features and disease progression of both conditions and offer an extensive review of relevant literature.

## Introduction

Rosai–Dorfman disease (RDD) was first described by Pierre-Paul Destombes in 1965, who reported four cases in his initial publication (1). Later, Rosai and Dorfman defined 34 cases and introduced the term sinus histiocytosis with massive lymphadenopathy, which was subsequently changed to RDD (2, 3). RDD is a rare benign disorder with a prevalence of 1/200,000 and is characterized by infiltration of large, pale histiocytes typically showing the microscopic characteristic feature of emperipolesis (4). RDD typically presents as painless cervical lymphadenopathy in children and young adults (3). The clinical course of RDD can be unpredictable with extranodal involvement in more than 40% of patients, even sometimes without associated lymphadenopathy (5). According to the revised classification of histiocytoses and neoplasms of the macrophage–dendritic cell lineages, RDD belongs to the R group of histiocytoses including familial, sporadic (classic nodal or extranodal), neoplasia-, or immunologic-associated RDD (6). The concomitant existence of RDD with lymphoproliferative disorders has been described in the literature, most often with non-Hodgkin lymphoma (7–11). We aim to report new insights into RDD by providing a detailed description of the first case of high-risk B-cell precursor acute lymphoblastic leukemia (ALL) in an adolescent who developed extranodal RDD with multiple osseous, hepatic, and dural involvements during the consolidation phase of treatment for ALL.

## Case report

A 14-year-old boy with high-risk pre-B-cell ALL, undergoing consolidation therapy with Children's Oncology Group (COG) AALL1131 protocol, presented with a 2-week history of recurrent and progressively intensifying bone pain. This pain was accompanied by swelling in both the upper and lower extremities, with his hands being the most noticeably affected in a pattern resembling dactylitis, along with the knees. Upon admission, no cervical lymphadenopathy was detected, and a detailed abdominal examination showed no abnormalities. However, a striking finding was the presence of extensive non-pitting edema involving the hands, knees, and legs. Given the clinical presentation, a bone marrow aspiration was performed to test for a possible leukemia relapse, which, reassuringly, revealed no measurable residual disease.

**Figure 1:**
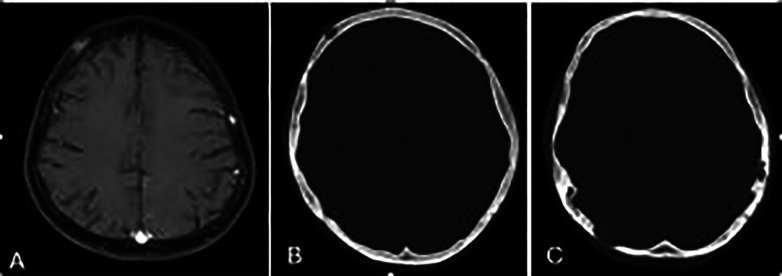
**(A)** Axial T1-weighted brain MR image showing enhancing lesions in the right frontal and left frontoparietal bones. **(B,C)** Axial bone window scan of the brain showing lytic lesions in the right frontal and occiput and left temporal bones with cortical erosion.

**Figure 2:**
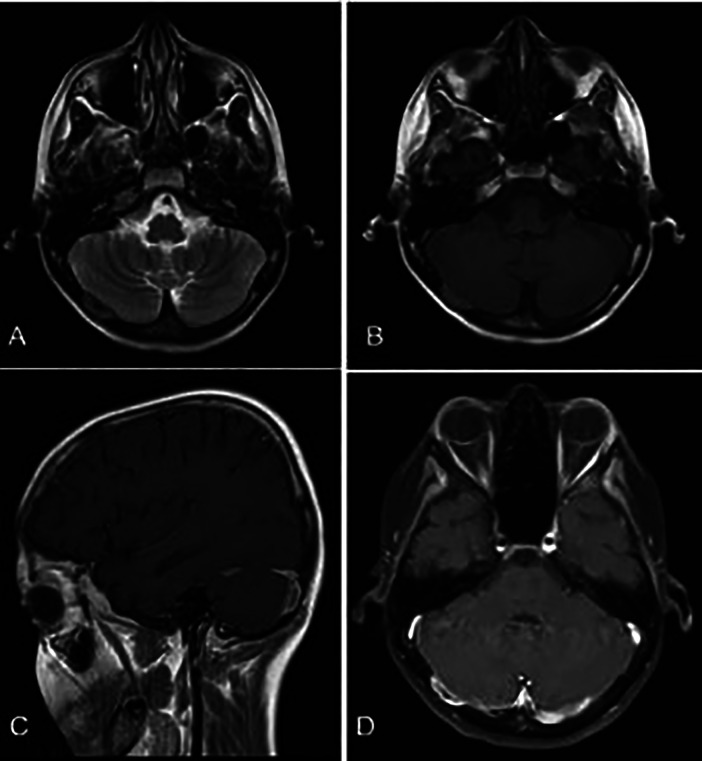
**(A,B)** Axial T1- and T2-weighted brain MR sequences. **(C)** Post-contrast sagittal and **(D)** axial T1-weighted images showing an isointense enhancing extra-axial mass posterior to the right cerebellar hemisphere in favor of dural involvement.

**Figure 3:**
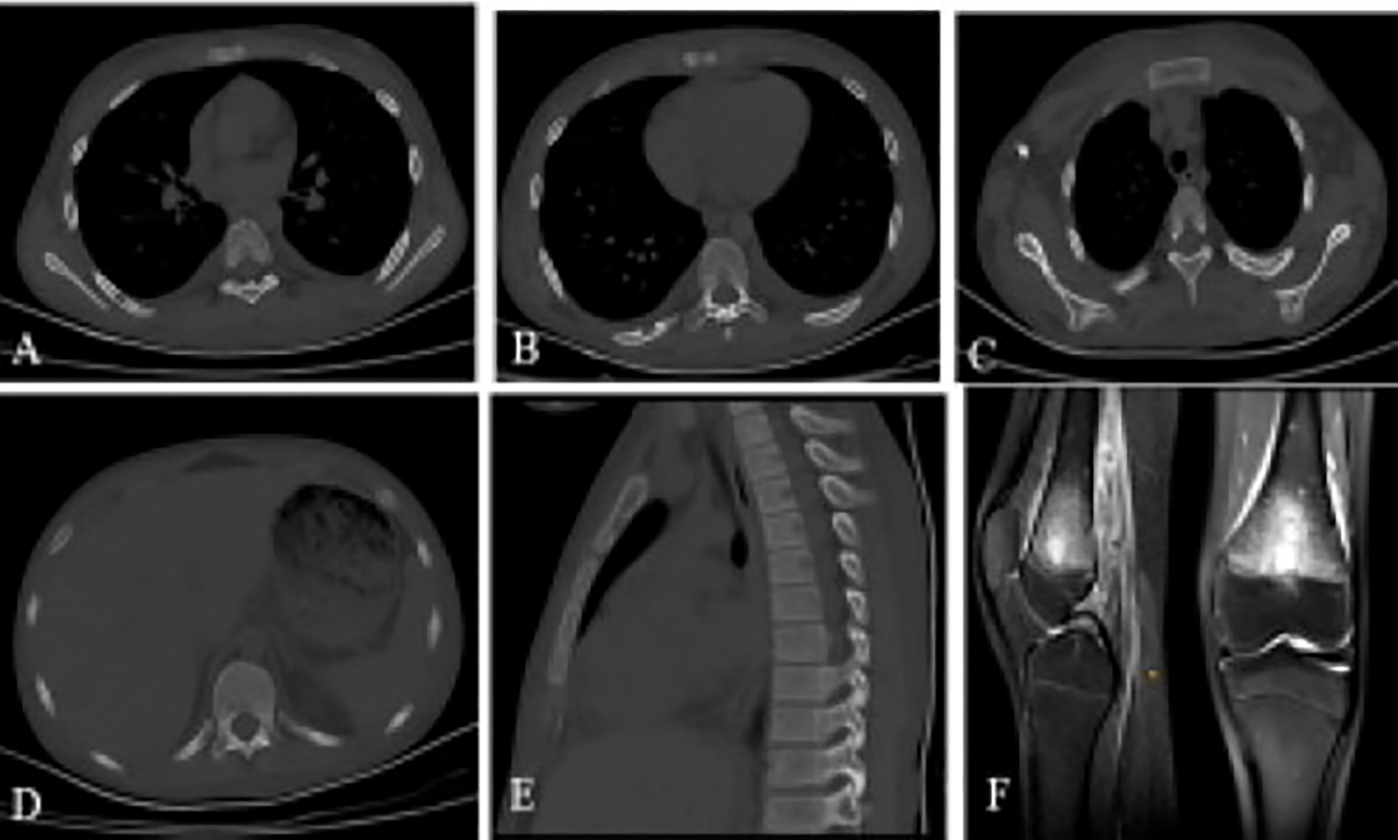
A chest CT scan showing **(A)** lytic lesion in manubrium of the sternum with cortical erosions, **(B)** T11 vertebral body, **(C** and **D)** MRI of the knee showing a lytic lesion in the distal femoral metaphysis with bone marrow and soft tissue edema, **(E–G)** Lytic lesions with cortical erosions in the right sixth and eighth ribs and medial aspect of the left scapula.

A brain CT scan identified lytic lesions with cortical erosion in the right frontal, occipital, and left temporal bones. Furthermore, a brain MRI scan showed enhancing lesions affecting the right frontal and left frontoparietal bones (Figure 1). In addition, post-contrast T1-weighted images showed an extra-axial enhancing isointense mass in the right occipital lobe posterior to the right cerebellar hemisphere, consistent with dural involvement (Figure 2). A chest CT scan showed lytic lesions in the ribs, left scapula, T11 vertebral body, and manubrium of the sternum with cortical erosions. The mediastinum and hilar regions were normal. MRI of a knee showed a lytic lesion in the distal femoral metaphysis with bone marrow and soft tissue edema (Figure 3). An abdominal ultrasound examination was remarkable for hypoechoic lesions throughout both liver lobes, with the largest measuring 10 × 11 mm in the right liver lobe. These lesions had not been detected in images acquired at the induction of chemotherapy for ALL.

**Figure 4:**
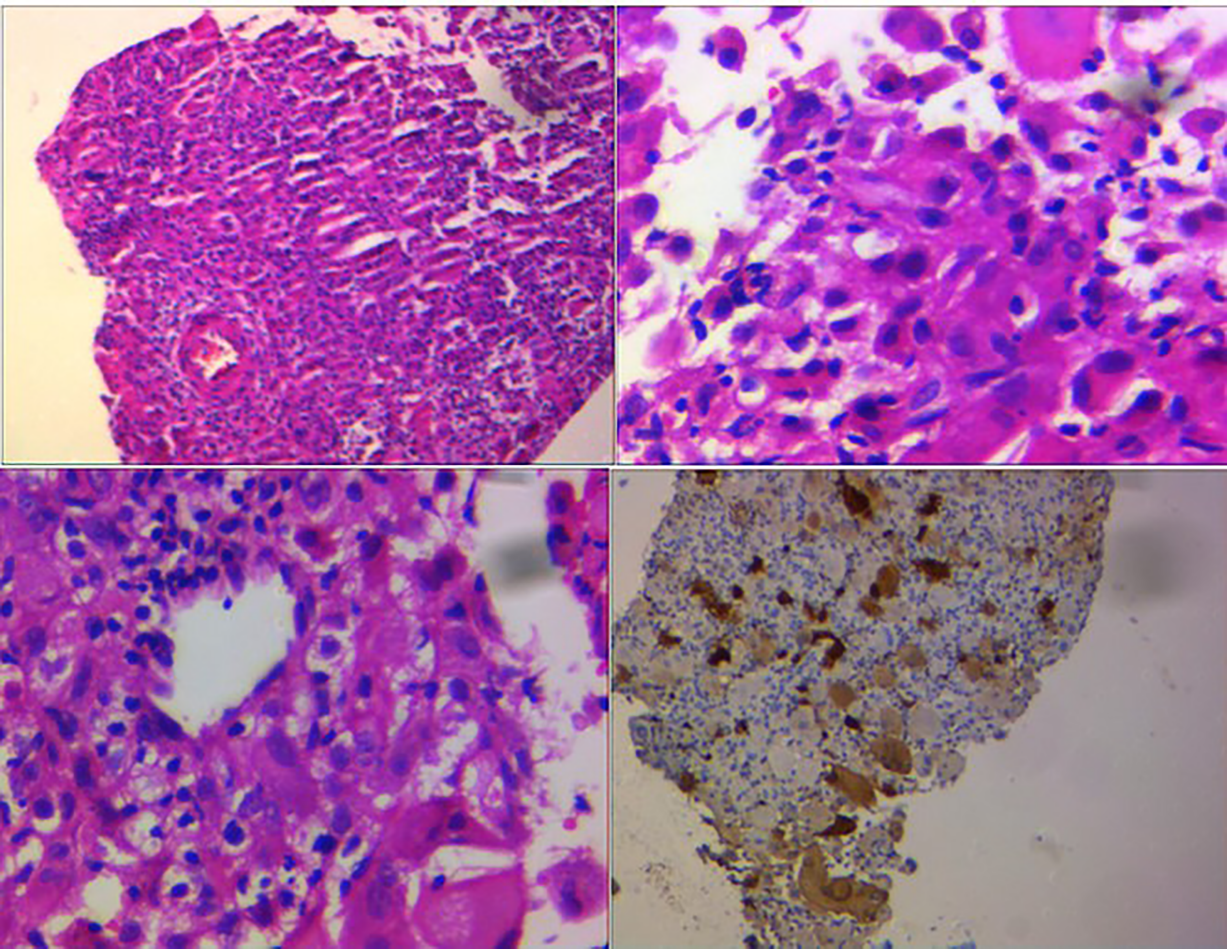
Large histiocytes with large oval nuclei and abundant eosinophilic cytoplasm admixed with lymphocytes and multinucleated inflammatory cells, some of which were engulfed by the histiocytes. This feature was interpreted as “emperipolesis.” IHC staining revealed positivity for S-100.

For further evaluation, a scapular bone biopsy was performed, in which histopathology revealed infiltration of large histiocytes with large oval nuclei and abundant eosinophilic cytoplasm, admixed with lymphocytes and multinucleated inflammatory cells, some of which were engulfed by the histiocytes. This phenomenon was interpreted as “emperipolesis.” Immunohistochemistry (IHC) staining showed positivity for S-100, cyclin D1, and CD68 in the histiocytes, while CD1a was negative, strongly suggesting RDD. A liver biopsy was also performed and demonstrated infiltration of large histiocytes with round vesicular nuclei and pink cytoplasm, along with phagocytosis of lymphocytes and neutrophils in portal spaces, confirming liver involvement in RDD (Figure 4).

During the interim maintenance phase of ALL treatment, the patient experienced persistent bone pain despite the administration of high-dose methotrexate. To address this challenge, a treatment regimen combining cladribine and ALL-directed therapy was initiated but demonstrated no significant symptom relief. While the MAPK pathway was not genetically analyzed, trametinib (0.025 mg/kg) was introduced on an empirical basis given its documented efficacy in non-Langerhans cell histiocytosis (non-LCH). Notably, this intervention resulted in marked clinical improvements, including a significant reduction in pain, diminished swelling of the extremities, and resolution of the skull lesions, as confirmed by follow-up MRI. The patient proceeded with the delayed intensification phase of the AALL1131 protocol, which included weekly administration of vincristine and doxorubicin. Subsequently, he required hospital admission due to an extended episode of febrile neutropenia. To further evaluate his condition, a bone marrow aspiration was conducted which indicated relapse with more than 5% lymphoblasts on multicolor flow cytometry. Nevertheless, the patient's clinical trajectory was marked by a disseminated fungal infection which resulted in death.

## Discussion

Histiocytic disorders of childhood have confused pediatric oncologists and pathologists over the years. RDD was classified as a benign non-LCH disorder for many years. According to the 5th edition of the World Health Organization classification of myeloid and histiocytic/dendritic neoplasms, RDD has been categorized as a histiocytic/dendritic neoplasm subsequent to a myeloid neoplasm to recognize its origin from common myeloid progenitors (12).

RDD classically presents with lymphadenopathy in children and young adults. The most common presentation of sporadic RDD is massive bilateral cervical lymphadenopathy followed by mediastinal, inguinal, and retroperitoneal localizations (13). Extranodal RDD has been identified in 40% of cases with the most frequent sites being the skin, bone, soft tissue, retro-orbit, upper respiratory tract, liver, pancreas, and lungs (3). Head and neck involvement has been reported in approximately 20% of cases which could be associated with extracranial nodal involvement (14). Bone involvement in RDD is rarely reported with approximately 10% of the cases associated with nodal involvement, nonetheless, primary bone involvement in the absence of lymphadenopathy occurs very rarely in 1% to 8% of the patients (3, 15).

The case presented in this case report demonstrated extensive involvement of all his skull bones accompanied by dural involvement, a condition that posed significant clinical challenges. Remarkable improvement was observed following the administration of trametinib, an innovative oral “mitogen-activated protein kinase” (MEK) inhibitor specifically utilized for RDD. This treatment highlights the potential of targeted therapies in addressing complex and rare conditions, offering new hope for patients with extensive disease manifestations (16, 17). Bone involvement in RDD is usually solitary with multifocality being extremely rare (18).

A systematic review of cases published between January 2000 and April 2015, utilizing the PubMed database, identified 108 patients with RDD involving the skeletal system. This review provides valuable insights into the clinical presentations, affected sites, and unique features of skeletal RDD. The average age of the patients diagnosed with skeletal RDD was 31.1 years (±19.8 years), with no pediatric cases identified, though three instances were reported in neonates. Primary RDD of the bone defined by the absence of lymphadenopathy throughout the disease course was observed in 74% of cases. Among these, 28 patients exhibited bone lesions exclusively, while others demonstrated concurrent involvement of additional organs such as the central nervous system (CNS), soft tissue, orbit, nasal cavity, muscle, or kidneys. Lesions were distributed across both axial and appendicular skeletal structures. Frequently affected sites included the cranium, facial bones, spine, sacrum, ribs, clavicle, sternum, scapula, humerus, radius/ulna, carpal bones, pelvis, femur, tibia/fibula, and tarsal bones. Notably, in 94% of cases, only one or two bones were involved, with a single patient presenting with eight affected bones. This wide anatomical distribution underscores the potential for RDD to impact nearly any skeletal region (19). The presented case exhibited lesions across various bones, including the skull, ribs, scapula, vertebrae, and sternum, with liver and dural involvement. Notably, the absence of nodal involvement makes this case unique, despite the extensive dissemination of the disease. The diagnosis was confirmed through tissue biopsies obtained from the scapula and liver. IHC played a pivotal role in substantiating the findings and ensuring diagnostic accuracy.

Isolated CNS involvement in RDD is an extremely rare occurrence, manifesting radiologically as dural-based extra-axial enhancing masses (20, 21). In the presented case, an isointense enhancing lesion was also observed in the posterior cerebellar hemisphere, which demonstrated a positive response to the RDD-directed treatment. This case underscores the importance of customized therapeutic strategies in addressing complex cases of RDD.

Hepatic involvement in RDD is an exceedingly rare occurrence, with an estimated incidence ranging from 1% to 5%. When present, it can manifest as single or multiple hepatic nodules or as diffuse hepatomegaly. These presentations are atypical and may pose diagnostic challenges due to their non-specific nature and overlap with other hepatic pathologies. Early recognition and accurate diagnosis are critical for guiding appropriate management strategies, as hepatic involvement in RDD is uncommon and often requires a multidisciplinary approach for optimal care (3, 22–24). A recent report from the University of Florida focused on a series of 11 adult patients with RDD who presented with gastrointestinal lesions. Among these patients, five were also found to have hepatic lesions (25).

RDD may exhibit associations with other histiocytic neoplasms, including Langerhans-cell histiocytosis (LCH), Erdheim–Chester disease, or malignant histiocytosis (6, 26). These conditions share overlapping pathological features, often involving an abnormal proliferation or accumulation of histiocytes, which can complicate diagnosis and management. While RDD is typically characterized by benign histiocytic proliferation, its coexistence with more aggressive or systemic histiocytic disorders necessitates careful clinical evaluation and tailored therapeutic approaches. Understanding the potential relationships between these entities is critical for accurate classification and optimizing treatment strategies.

Systemic forms of RDD have been observed either concurrently with or subsequent to various types of non-Hodgkin's lymphomas. This association highlights a potential overlap in the pathophysiological mechanisms or shared immunological pathways between these conditions. While RDD is a rare histiocytic disorder characterized by the excessive proliferation of histiocytes, its co-occurrence with lymphoproliferative malignancies such as non-Hodgkin's lymphoma suggests a need for further investigation into their relationship (7–11). A review of the existing literature has identified eight documented cases in which RDD has been found to coexist with Hodgkin lymphoma in adult patients. This finding highlights a rare but notable overlap between the two conditions (27, 28).

Ambati et al. reported the case of a 13-year-old boy diagnosed with pre-B cell ALL who developed RDD 8 months after a bone marrow transplant (29). In 1991, the Histiocyte Society conducted a survey among its members to document cases of LCH associated with malignancies. The findings revealed 13 cases where LCH occurred in conjunction with acute leukemia. Among these, five patients with ALL developed LCH either during chemotherapy or within 6–12 months after completing treatment for ALL. Additionally, eight cases involved the development of acute myeloid leukemia (AML) following treatment for LCH. These observations highlight a notable association between LCH and hematologic malignancies, warranting further investigation into potential underlying mechanisms and clinical implications (30). A retrospective analysis of T-cell ALL (T-ALL) cases registered in the ALL-BFM trials from 1981 to 2001 revealed that 6 out of 971 patients, accounting for 0.4%, developed either hemophagocytic lymphohistiocytosis (HLH) or LCH. This finding highlights the rarity of these complications in the context of T-ALL and underscores the importance of vigilance for such conditions during treatment and follow-up (31).

A rare case of T-ALL was reported in a 7-year-old female patient who presented with cervical lymphadenopathy 3 years after she had completed chemotherapy for leukemia. Following her initial workup, she was diagnosed with RDD. This case highlights the complexity of managing pediatric leukemia survivors, particularly when secondary conditions such as RDD emerge post-treatment (32). Only a single case of biphenotypic acute leukemia has been documented in a 4-year-old boy, emerging approximately 15 months following the diagnosis of RDD. This case highlights the potential for complex hematological disorders to develop in the context of pre-existing conditions (33).

## Conclusion

This case represents the first recorded occurrence of RDD presenting simultaneously with B-cell ALL during chemotherapy. Remarkably, it involved multiple bones, the dura mater, and the liver without affecting the lymph nodes, rendering it particularly distinctive. The observed link between RDD and malignant hematologic neoplasms appears non-coincidental, underscoring the importance of increased clinical awareness. This report emphasizes the necessity of closely monitoring patients with hematologic malignancies for unusual manifestations, such as organ involvement or lymphadenopathy inconsistent with leukemia, both during and post-chemotherapy. Such observations warrant further investigation for rare comorbidities such as RDD given their potential concurrence. The intersecting and evolving clinical courses of these conditions present notable challenges in management, highlighting the value of a multidisciplinary approach and vigilant observation to ensure the best possible patient outcomes.

## Data Availability

The original contributions presented in the study are included in the article/Supplementary Material, further inquiries can be directed to the corresponding author.
